# Transcriptomic and metabolomic insights into the synergistic effects of resveratrol and β-hydroxy-β-methylbutyric acid on hepatic function under varying protein diets in Tibetan sheep

**DOI:** 10.3389/fnut.2025.1614114

**Published:** 2025-07-08

**Authors:** Yu Zhang, Kaina Zhu, Fengshuo Zhang, Zhenling Wu, Shengzhen Hou, Linsheng Gui

**Affiliations:** College of Agriculture and Animal Husbandry, Qinghai University, Xining, Qinghai, China

**Keywords:** resveratrol, β-hydroxy-β-methylbutyric acid, Tibetan sheep, liver, transcriptome, metabolome

## Abstract

**Background:**

Increasing evidence indicated resveratrol (RES) and β-hydroxy-β-methylbutyric acid (HMB) regulated several biological processes via modulating gene expression. This study employed transcriptomic and metabolomic analyses to investigate the impact of RES and HMB supplementation, in combination with varying dietary protein levels on hepatic immunity, antioxidant capacity, and morphology in Tibetan sheep.

**Methods:**

Two treatments (with or without RES and HMB supplementation) and two dietary protein levels (12% vs. 14% of the basal diet) were tested according to a 2 × 2 factorial arrangement within a Latin square design. A total of 120 healthy two-month-old male Tibetan lambs (16.87 ± 0.31 kg) were randomly allocated for 90-day feeding experiment, with the following treatments: low-protein basal diet without (L group) or with (L-RES + HMB group) supplemental 1.50 g/d RES and 1.25 g/d HMB, and high-protein basal diet without (H group) or with (H-RES + HMB group) supplemental 1.50 g/d RES and 1.25 g/d HMB.

**Results:**

The results indicated that the liver tissue structure was predominantly normal in the H-RES + HMB group, devoid of central vein congestion. The catalase (CAT) activity and total antioxidant (T-AOC) capacity were significantly increased when fed the 14% protein diet (*p* < 0.05). The superoxide dismutase (SOD) and CAT activities of sheep fed supplementary-treated diets were significantly increased than the basal diet (*p* < 0.05). Immunoglobulin M (IgM) level and tumor necrosis factor-alpha (TNF-*α*) activity in the H-RES + HMB group were significantly increased than those in the H and L groups (*p* < 0.05), whereas interleukin-1 beta (IL-1β) levels were significantly lower (*p* < 0.05). A total of 4,236 differentially expressed genes (DEGs) were identified, including 3,503 upregulated genes and 733 downregulated genes, which were categorized into immune-related KEGG signaling pathways. Metabolomic analysis identified that compared to L group, the abundance of 918 metabolites were significantly changed in H-RES + HMB group including 829 upregulated and 89 downregulated. Those differential metabolites enriched in KEGG pathways primarily related to immunity and antioxidation.

**Conclusion:**

Dietary protein level and RES/HMB supplementation exhibited positively interaction effect on immunity and antioxidant capacity. The 14% protein diet with RES and HMB improved the hepatic function through modulating the gene and metabolite in Tibetan sheep.

## 1 Introduction

The liver serves as a pivotal metabolic organ in animals, engaging in a multitude of metabolic processes (1), including fat metabolism, protein synthesis and detoxification (conversion of ingested toxins into non-toxic or less toxic substances via various enzymatic reactions, including oxidation, reduction, hydrolysis, and conjugation through enzyme systems such as cytochrome P450, followed by excretion). Furthermore, the liver plays a crucial role in gluconeogenesis, utilizing non-carbohydrate substrates to produce glucose through key enzymatic reactions during fasting or starvation, thus maintaining blood glucose levels (2). The nutrients absorbed through the gastrointestinal tract are transported to the liver via the portal vein, where they undergo further metabolism and regulation. This influences the animal’s energy balance and nutritional status (3).

Resveratrol (RES) was a natural polyphenol compound in grape skins, wine and peanuts, and was known for its anti-inflammatory and anti-oxidant effects (4). RES mitigated oxidative stress by modulating the Nrf2 phosphorylation/nuclear translocation in the HepG2 cells, thereby alleviating the hepatitis B virus-related liver damage (5). RES improved the expression level of lncRNA Snhg6 in the exosomes, which were involved in the regulation of hepatic stellate cell activation (6). The plasma triglyceride, free fatty acids, and serum amyloid A concentrations and lactate dehydrogenase activities markedly decreased when supplemented RES, contributing to improvement of liver function in high-fat fed cat (7). As a metabolite of the essential amino acid leucine, β-hydroxy-β-methylbutyric acid (HMB) was recognized for its pivotal role in promoting protein synthesis, inhibiting protein degradation, enhancing exercise capacity, facilitating growth, and boosting immunity (8). 0.10% HMB supplementation in broilers inhibited the hepatic fat deposition via regulating the relative abundance of *Bacteroidetes* (9). Via regulating AMP-activated protein kinase *α* (AMPK) and Toll-like receptor 4 (TLR4) signaling pathways, dietary HMB supplementation participated in hepatic energy metabolism and ameliorated liver injury in the lipopolysaccharide (LPS)-challenged piglets (10).

Tibetan sheep are a valuable livestock species local to the Qinghai-Tibet Plateau, which are well-adapted to harsh conditions such as high altitudes, low oxygen levels, and limited food resources (11). Previously, the dietary RES and HMB alone or in combination affected the hepatic antioxidant capacity, immune response, and glycolytic activity (12). However, the interaction effect of RES/HMB supplementation and dietary protein levels on hepatic function of Tibetan sheep remain unclear. Therefore, the objective of this study was to determine the effects of using RES and HMB in varying protein diets on liver morphology, antioxidant ability, immune response using the transcriptomic and metabolomic, which might help to identify a possible mechanism of RES and HMB for promoting health status in Tibetan sheep.

## 2 Materials and methods

### 2.1 Animals, experimental design, and diets

A total of 120 2-month-old healthy male lambs (16.87 ± 0.31 kg) were selected and randomized into four treatments: 12% protein level (L group), 14% protein level (H group), 1.5 g/d resveratrol + 1.25 g/d HMB under 12% protein level (L-RES + HMB group), and 1.5 g/d resveratrol + 1.25 g/d HMB under 14% protein level (L-RES + HMB group). The experiment was conducted for a total of 100 d, including 10 days of pre-feeding and 90 days of the trial period. The feed consisted of roughage (oat green hay and oat silage at a 1:1 dry matter ratio) and concentrate, and the ratio of concentrate to roughage was 7:3. The RES (purity >99%) used in this experiment was purchased from Xi’an grass plant technology Co., Ltd. (Xi’an, China). HMB (purity >99%) was purchased from TSI Group Co., Ltd. (Shanghai, China). Both RES and HMB were firstly added to the premix, and then directly mixed with concentration. The experimental sheep were fed twice a day at 08:00 and 17:00, and had *ad* libitum access to diets and clean drinking water. The formulation and nutritional content of the diet are shown in Table 1. At the end of this period, five Tibetan sheep of similar body condition from each group were selected for slaughter.

**Table 1:** Composition of the basic diet.

Items	L-CP	H-CP
Ingredient (%)		
Corn	58.3	51.50
Soybean meal	1.00	2.00
Rapeseed meal	7.00	12.80
Cottonseed meal	2.00	2.00
Palm meal	25.00	25.00
Nacl	1.00	1.00
Limestone	1.00	1.00
Baking soda	0.10	0.10
Premix^1^	4.60	4.60
Total	100.00	100.00
Nutrient levels^2^		
Digestibility/(MJ·kg^-1^) DE	12.84	12.71
Crude protein (%)	12.13	14.27
Ether extract (%)	3.44	3.29
Crude fiber (%)	11.05	11.64
Neutral deterzent fiber (%)	26.04	26.70
Acid detereent fiber (%)	19.11	19.97
Ca (%)	0.80	0.84
P (%)	0.35	0.4

^1^Premix provides Cu 18 mg, Fe 66 mg, Zn 30 mg, Mn 48 mg, Se 0.36 mg, I 0.6 mg, Co 0.24 mg, VA 24,000 IU, VD 4,800 IU, and VE 48 IU per kg of diet. ^2^Digestive energies are calculated values, and the rest are measured values.

### 2.2 Liver morphology

The liver tissue fixed in 4% polyformaldehyde was dehydrated using a gradient of ethanol solutions (Beijing BOAOtoda Technology Co., Ltd., Beijing, China) and then cleared with xylene (Shanghai BOAOtoda Denuo chemical Co., Ltd., Shanghai, China). After dehydration and clarification, the liver tissue was embedded in paraffin and sliced into 3–4 μm thick sections. After drying, dewaxing, and hydration, hematoxylin and eosin (HE) staining was performed, and the morphological changes in the liver tissue were observed under a microscope.

### 2.3 Biochemical determination

The liver sample (approximately 1.00 g) was mixed with 9 mL pre-cooled phosphate-buffered saline (PBS, Thermo Fisher Scientific, MA, United States). The homogenate was centrifuged at 4°C and 3,000 × g for 20 min, and the supernatant was stored at −80°C for biochemistry analysis. The antioxidant indicators including total antioxidant capacity (T-AOC), catalase (CAT), superoxide dismutase (SOD), glutathione peroxidase (GSH-PX), and malondialdehyde (MDA), immune indicators including interleukin-1 beta (IL-1β), interleukin-6 (IL-6), immunoglobulin A (IgA), immunoglobulin G (IgG), immunoglobulin M (IgM), and tumor necrosis factor-alpha (TNF-*α*), and glycolytic indicators including lactate dehydrogenase (LDH), glucose (GLU), liver/muscle glycogen (L/M), hexokinase (HK), pyruvate kinase (PK), creatine kinase (CK), lactate (LA), malic dehydrogenase (MD), and succinate dehydrogenase (SDH) were measured using the enzyme-linked immunosorbent assay (ELISA) kits (Jiangsu Meimian Industrial Co., Ltd., Jiangsu, China).

### 2.4 Transcriptome analysis

Liver tissue samples were preserved on dry ice and sent to Gene Denovo Biotechnology Co., Ltd. (Guangzhou, China), where total RNA extraction was performed using the TRIzol method (TIANGEN Biotech Co., Ltd., Beijing, China). The RNA quality was subsequently assessed using an Agilent 2,100 Bioanalyzer (Agilent Technologies, CA, United States) and agarose gel electrophoresis were employed to assess RNA quality. The RIN values ranged from 7.4 to 8.2. Subsequently, oligonucleotide (dT) beads were utilized for the enrichment of eukaryotic messenger ribonucleic acid (mRNA), with a Ribo-Zero™ Magnetic Kit (Epicentre, WI, United States) employed for the removal of ribosomal RNA (rRNA). Enriched mRNA samples were suspended in a fragmentation buffer to generate short fragments, after which cDNA was generated via reverse transcription using random primers. Subsequently, DNA polymerase I, RNase H, and dNTPs were utilized to initiate second-strand cDNA synthesis (Qiagen, Venlo, Netherlands). This was followed by the purification of the cDNA using a QiaQuick PCR extraction kit (Qiagen, Venlo, Netherlands). Subsequent to the isolation process, the cDNA was subjected to a series of molecular biology techniques, including end repair, poly-adenylation, and Illumina sequencing adapter ligation. Agarose gel electrophoresis was subsequently employed for the selection of ligation product size, followed by PCR amplification and sequencing with the Illumina HiSeq2500 platform (Illumina, Inc., San Diego, CA, United States).

Per the standard data processing protocol employed by Gene Denovo Biotechnology Co., Ltd. (Guangzhou, China), the original data obtained from sequencing were filtered to obtain high-quality clean reads. Clean reads were then mapped to reference genome (Genome: Oar_Version 3.1) using HISAT2^1^ (version hisat2-2.0.4). Subsequently, the HISAT2 software (version 2.2.1) was used for sequence alignment to obtain specific sequence information and perform genomic localization analysis (13).

The threshold for screening DEGs was set at FDR < 0.05 and |log_2_FC| > 1, where fold change (FC) represented the difference in gene expression between different treatment groups. The FDR correction was calculated using the Benjamini-Hochberg method. The DEGs were functionally annotated and pathway enrichment analysis was performed using the Gene Ontology (GO) (14) and Kyoto Encyclopedia of Genes and Genomes (KEGG) (74) databases. *p <* 0.05 indicated the enrichment of related genes in different biological pathways.

### 2.5 Weighted gene co-expression network analysis

The RNA-seq data were pre-processed to filter out genes with low expression levels and to normalize the expression values. The processed gene expression matrices were then used for WGCNA (15). Correlations were emphasized and neighbor-joining matrices were created by calculating Pearson correlation coefficient matrices between all gene pairs and raising these correlation coefficients to a power (soft threshold) (16). Modules of highly correlated genes were identified using a hierarchical clustering approach and a dynamic tree-cutting algorithm. Characteristic genes were calculated for each module, and the relationship between modules and traits was analyzed. The association between modules and traits was investigated in Tibetan sheep liver with the addition of RES and HMB to the diet at different protein levels. Genes (GS) and modules (MM) were identified to assess the importance of individual genes within each module and their relationship to module trait genes. Genes within the important modules were further analyzed for functional enrichment to gain insight into the biological processes and pathways regulated by the co-expressed genes.

### 2.6 Verification of DEGs

To verify the results of transcriptome sequencing, a number of DEGs were randomly selected. Primers were designed using Primer 5.0 software (PRIMER-E Ltd., Plymouth, United Kingdom), and the expression levels of these genes were verified using the qRT-PCR method. Total RNA was extracted from liver of using the TRIZOL method (TIANGEN Biotech Co., Ltd., Beijing, China). Total RNA extracted from each sample was reverse transcribed into cDNA, which was used as a template for qRT-PCR (primer sequences are shown in Table 2). Glyceraldehyde-3-phosphate dehydrogenase (GAPDH) as the internal reference. The reaction system, program, and method used for calculating the relative expression levels of genes based on the protocol reported by Li et al. (17). Relative RNA expression was calculated using the 2^−ΔΔCT^ method.

**Table 2:** qRT-PCR primer sequences.

Name	Primer sequence (5’-3’)	Tm (°C)	Product length
GLYAT	F-CCTGATGGACCAGACGGGAGAG R-GGTAGATGGCATGGGTGACAAGC	60.0	86bp
IL1R1	F-CCGTTCGTGTCCTCTCATCACAAG R-ACTTCAGCAGGAACAAACCAGAGC	60.0	141bp
TNC	F-CGTTACCGCCTCAACTACAGTCTTC R-TTGCTCTTGTGTCTGCCTTTCTCTG	60.0	149bp
TUBA1C	F-CTCGCCTGGACCACAAGTTTGAC R-TCGGCTCCAACCTCCTCATAATCC	60.0	148bp
PCK1	F-TACTTTGCGGCTGCGTTTCCC R-CACCCACACACTCCACTTTCCATC	60.0	94bp
GABRG1	F-GGAGGGAAGGGAGGATACACATACG R-AAACCAGATTGAACAGGGCAAAAGC	60.0	93bp
ENPP3	F-AAACTCTCAGGATGCCGCTTTGG R-CAGTCTGGGACAGTGGGAGGAAG	60.0	85bp
AIF1L	F-AGAAGATGATCTCGGAGGTGACAGG R-AGCGTTTCCCCAGCATCATGTTC	60.0	84bp

### 2.7 Metabolomics analysis

Liver tissue samples preserved on dry ice were sent to Gene Denovo Biotechnology Co., Ltd. (Guangzhou, China). The samples were freeze dried in the same proportions, after which 1,000 μL of methanol stored at −20°C was added to the lyophilized powder. The samples were then subjected to centrifugation at 12,000 rpm for 10 min at 4°C. Subsequently, a 450 μL aliquot of the clarified serum was subjected to vacuum concentration, resulting in the dissolution of the sample within 150 μL of a 2-chlorobenzalanine (4 ppm) 80% methanol solution. This solution was then filtered through a 0.22 μm membrane to ensure the removal of any particulate matter. A 20 μL aliquot of each sample was utilized for quality control (QC) analysis, while the remaining samples were allocated for liquid chromatography-mass spectrometry (LC–MS) analysis. The samples were subjected to analysis using a UHPLC-QE Orbitrap/MS system, which comprised an Agilent 1,290 Infinity LC ultra-high-performance liquid chromatography system (Agilent Technologies, Inc., Santa Clara, CA, United States) and an AB Triple TOF 6600 mass spectrometer (SCIEX, Framingham, MA, United States). This configuration was employed for the separation and acquisition of spectra for the samples. The original data were converted to the MZML format through the utilization of Proteo Wizard software (ProteoWizard Development Team, USA) (18, 19). The XCMS Online (Scripps Research, United States) was employed for peak identification, retention time correction, peak alignment, and the extraction of peak areas. The XCMS (20) program was also utilized to obtain a data matrix, which was then annotated with mass spectral database peaks using the OSI-SMMS software (version 4.2) (Object Security, LLC, San Jose, CA, United States). Normalized data were imported into SIMCA^®^-P 16.0 (Sartorius Stedim Data Analytics, Sweden) for multivariate statistical analysis. Significantly different metabolites were screened using the t-test based on VIP ≥ 1 and *p <* 0.05 as the screening thresholds (21). The hypergeometric test was employed to identify significantly enriched pathways, with an FDR correction threshold of 0.05.

### 2.8 Transcriptomics and metabolomics correlation analysis

The identified DEGs were combined with differentially abundant metabolites (DAMs), and the two-dimensional orthogonal partial least squares (O2PLS) method was used to identify overlapping metabolic pathways and detect the immune-related pathways represented in the DEG and DAM datasets (22). Pearson’s correlation coefficients were used for the comprehensive analysis of metabolomics and transcriptomics data, and Cytoscape 3.8.9 (Cytoscape Consortium, United States) was employed to visualize gene and metabolite pairs with shared pathways (Pearson’s correlation coefficient >0.995 and *p <* 0.05). Metabolite transcription network analysis was conducted on gene–metabolite pairs with a Pearson’s correlation coefficient >0.5.

### 2.9 Statistical analyses

Results are expressed as means ± standard deviations. Experimental data were processed using Microsoft Excel 2019 (Microsoft Corporation, Redmond, WA, United States) and then subjected to two-factor variance analysis using the SPSS 26.0 statistical analysis software (IBM Corp., Armonk, NY, United States), with Duncan’s method employed for multiple comparisons. Significance thresholds were determined as follows: *p <* 0.01, highly significant difference; *p <* 0.05, significant difference; *p* > 0.05, no significant difference.

## 3 Results

### 3.1 Effect on liver morphology

**Figure 1:**
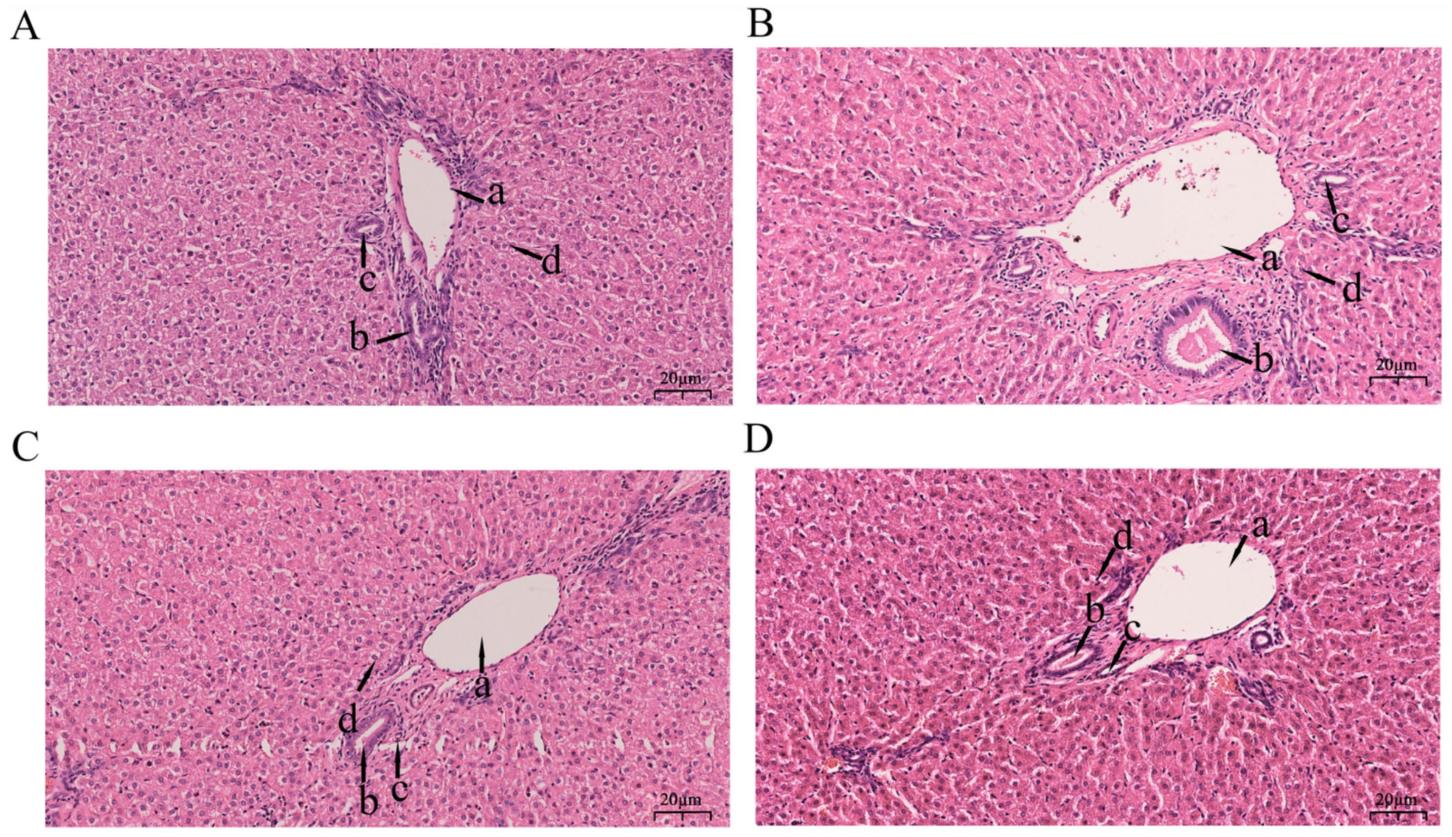
Stained section of liver tissue of Tibetan sheep fed with different levels of resveratrol and HMB. Panel **(A)** is the 12% basal diet (L Group), Panel **(B)** is the 12% basal diet + resveratrol (1.5 g/day) + β-hydroxy-β-methylbutyrate (1.25 g/day) (L-RES-HMB Group), Panel **(C)** is the 14% basal diet (H Group), and Panel **(D)** is the 14% basal diet + resveratrol (1.5 g/day) + β-hydroxy-β-methylbutyrate (1.25 g/day) (H-RES-HMB Group). HE staining, 2001×. a: Interlobular vein; b: Interlobular bile duct; c: Interlobular artery; d: Hepatic sinusoid.

As shown in Figure 1, hepatic tissues across all groups (L, L-RES + HMB, H, H-RES + HMB) generally maintained normal architecture, with hepatocytes radially arranged around central veins. The L group exhibited central vein congestion. The L-RES + HMB group showed red blood cells within central veins and dilated sinusoids. Both H and H-RES + HMB groups displayed mildly irregular tissue structures, though overall hepatic conditions were comparable. The H group featured red blood cells in central veins with dilated and congested sinusoids. In contrast, the H-RES + HMB group retained largely normal architecture without central vein congestion. Notably, both RES + HMB groups (L and H) showed red blood cells in central veins and dilated sinusoids.

### 3.2 Effect on antioxidant parameters in the liver

Among the animals receiving different amounts of dietary crude protein (CP), the CAT activity and T-AOC were significantly higher (*p <* 0.05) under the low CP diet condition (12% CP, LCP) than under the high CP diet condition (14% CP, HCP), while MDA activity was significantly lower. Among untreated animals and animals treated with RES and HMB, the non-RES + HMB group (N-RES + HMB group) exhibited significantly higher SOD and CAT activities than the RES + HMB group (*p <* 0.05). Conversely, the RES + HMB group displayed a significantly lower MDA activity (*p <* 0.001) and higher GSH-PX activity (*p <* 0.05) than the non-RES + HMB group. An interaction effect between protein content and RES and HMB supplementation was observed, leading to differences in SOD and MDA activities. Specifically, the H-RES + HMB group demonstrated significantly higher SOD activity (*p <* 0.05) than the other groups, while the L group exhibited significantly lower MDA activity (*p <* 0.001) than the other group. No significant effects on other antioxidant indicators were observed (Table 3).

**Table 3:** The impact of adding RES and HMB at different protein levels on the antioxidant activity in the liver of Tibetan sheep.

Items	Groups	SOD (pg/mL)	CAT (ng/L)	GSH-PX (pmol/mL)	AOC (u/mL)	MDA (pg/mL)
Protein Levels	L CP	106.25 ± 3.86^B^	167.65 ± 3.68^a^	47.00 ± 2.87^b^	12.31 ± 1.40^a^	3.23 ± 0.39^A^
	H CP	143.95 ± 14.61^A^	151.39 ± 3.24^b^	61.78 ± 6.29^a^	11.77 ± 0.59^b^	3.90 ± 0.42^B^
Additive Dosage	N-RES + HMB	180.64 ± 4.26^a^	170.15 ± 15.15^a^	61.52 ± 9.76^b^	9.87 ± 0.538	8.21 ± 0.68^B^
	RES + HMB	172.37 ± 7.60^b^	157.40 ± 7.65^b^	76.28 ± 5.79^a^	10.72 ± 0.24	4.13 ± 0.44^A^
*Protein* × *additives*	L	125.10 ± 22.45^A^	152.04 ± 13.49	55.45 ± 9.22	12.04 ± 1.03	3.74 ± 0.42^A^
	H	175.92 ± 7.38^B^	163.77 ± 13.04	68.89 ± 10.83	10.29 ± 0.59	5.95 ± 2.21^B^
	L-RES + HMB	138.13 ± 39.93^A^	168.01 ± 12.16	55.29 ± 10.51	11.09 ± 1.63	6.20 ± 2.55^B^
	H-RES + HMB	158.16 ± 18.63^C^	149.80 ± 9.98	69.03 ± 9.56	11.24 ± 0.69	4.03 ± 0.42^B^
*P*-value	CP Level	0.000	0.049	0.002	0.001	0.000
	RES-HMB	0.009	0.005	0.002	0.708	0.000
	RES-HMB × CP level	0.000	0.337	0.998	0.113	0.000

“Protein Levels” indicates ration protein level (12% protein and 14% protein) and ‘RES-HMB’ indicates addition, HMB (1.25 g/day) plus RES (1.50 g/day). “RES-HMB × CP level” indicates the interaction of RES and HMB with ration protein level. Within the same column, data without letters or with the same letters indicate no significant difference (*p >*0.05), different letters indicate significant difference (*p <*0.05), and different uppercase letters in the same column indicate extremely significant differences between groups (*p <*0.01), lowercase letters define significant differences (*p <*0.05). Glutathione peroxidase (GSH-PX), Superoxide dismutase (SOD), Total antioxidant capacity (T-AOC), Catalase (CAT), Malondialdehyde (MDA).

### 3.3 Effect on the immune activity in the liver

IgG and IgM levels in the HCP group (14% protein) were significantly higher than those in the LCP group (12% protein) (*p <* 0.001). Meanwhile, TNF-α and IL-6 levels were significantly lower in the HCP group (*p <* 0.05). Therefore, the protein content affected the activities of immune indicators. Regarding the effect of RES and HMB supplementation, the N-RES + HMB group exhibited significantly higher IgA and IgM activities (*p <* 0.001) and significantly lower TNF-α activity (*p <* 0.05) than the RES + HMB group. The RES + HMB group also displayed a significantly lower IL-1β (*p <* 0.001) and IL-6 activity (*p <* 0.05) than the N-RES + HMB group. Protein levels and RES and HMB supplementation exerted an interaction effect on IgG, IgM, TNF-α, and IL-1β levels. The HCP group had significantly higher IgG activity (*p <* 0.05) than the other groups, while the H-RES + HMB group had significantly higher IgM activity (*p <* 0.001) and lower IL-1β activity (*p <* 0.001) than the other groups. Additionally, the TNF-α activity in the H-RES + HMB group was significantly lower than that in the L-RES + HMB group (*p <* 0.001) (Table 4).

**Table 4:** The impact of adding RES and HMB at different protein levels on immune activity in the liver of Tibetan sheep.

Items	Groups	IgA (Mg/mL)	IgG (Mg/mL)	IgM (Mg/mL)	TNF-α (ng/L)	IL-1β (ng/L)	IL-6 (ng/L)
Protein Levels	L CP	14.52 ± 0.83	588.88 ± 43.21^B^	6.56 ± 0.29^B^	1000.76 ± 74.94^a^	95.70 ± 3.58	155.34 ± 40.10^a^
	H CP	10.27 ± 0.47	669.45 ± 69.61^A^	17.28 ± 0.55^A^	665.19 ± 48.78^b^	48.67 ± 1.89	89.27 ± 15.38^b^
Additive Dosage	N-RES + HMB	14.57 ± 1.43^A^	1062.04 ± 35.71	14.54 ± 0.58^A^	671.92 ± 19.10^b^	77.27 ± 4.71^A^	105.84 ± 29.2^a^
	RES + HMB	8.67 ± 0.62^B^	933.33 ± 19.64	14.21 ± 0.66^B^	700.76 ± 109.17^a^	63.82 ± 5.74^B^	50.12 ± 7.73^b^
*Protein* × *additives*	L	12.40 ± 2.35	637.22 ± 69.54^a^	11.93 ± 5.74^B^	832.98 ± 188.68^B^	68.83 ± 25.26^B^	117.59 ± 40.10
	H	11.62 ± 3.31	1010.55 ± 75.52^b^	14.37 ± 0.60^B^	686.34 ± 74.17^B^	70.54 ± 8.67^C^	77.98 ± 35.69
	L-RES + HMB	14.54 ± 1.08	872.77 ± 261.27^c^	10.55 ± 4.28^C^	836.34 ± 182.92^A^	85.17 ± 10.60^A^	127.06 ± 40.71
	H-RES + HMB	9.47 ± 0.99	775.00 ± 153.00^c^	15.74 ± 1.73^A^	682.98 ± 80.55^B^	56.25 ± 9.01^D^	69.69 ± 23.71
*P-*value	CP Level	0.116	0.000	0.000	0.001	0.477	0.005
	RES-HMB	0.000	0.477	0.000	0.001	0.000	0.001
	RES-HMB × CP level	0.097	0.016	0.000	0.000	0.000	0.694

Immunoglobulin A (IgA), Immunoglobulin G (IgG), Immunoglobulin M (IgM), Tumor necrosis factor-α (TNF-α), Interleukin-1 beta (IL-1β), Interleukin 6 (IL-6). Data in the same column with the same or no lower case letters indicate nonsignificant differences (*p* > 0.05) and data with different lower case letters indicate significant differences (*p* < 0.05).

### 3.4 Effect on glycolysis in the liver

The activity of hexokinase and pyruvate kinase in the LCP group (12% protein) was significantly higher than that in the HCP group (14% protein) (*p <* 0.05). Meanwhile, the activity of LDH in the HCP group was significantly higher than that in the LCP group (*p <* 0.001), and the activity of malate dehydrogenase and SDH was significantly lower (*p <* 0.001). Therefore, the protein content affected the activities of hepatic glycolytic enzymes. Regarding the effect of RES and HMB supplementation, the N-RES + HMB group had a significantly higher activity of pyruvate kinase than the RES + HMB group (*p <* 0.001) and a significantly lower activity of SDH (*p <* 0.001). Meanwhile, the N-RES + HMB group had a significantly lower activity of lactate than the RES + HMB group (*p <* 0.05) and a significantly higher activity of malate dehydrogenase (*p <* 0.05). Protein levels and RES and HMB supplementation exhibited an interaction effect on LDH, glucose, hexokinase, and SDH activities, with the H-RES + HMB group showing significantly higher LDH and hexokinase activities (*p <* 0.05) and SDH activity (*p <* 0.001) than the other groups. Meanwhile, the activity of glucose in the H-RES + HMB group was significantly higher than that in the L-RES + HMB group (*p <* 0.05) (Table 5).

**Table 5:** The impact of adding RES and HMB at different protein levels on glycolysis in the liver of Tibetan sheep.

Items	Groups	LDH (U/L)	GLU (mmol/L)	MG (mg/L)	HK (ng/L)	PK (ng/L)	LA (ug/l)	MDH (U/L)	SDH (U/mL)
Protein Levels	L CP	40.62 ± 1.23^B^	585.89 ± 6.54	126.65 ± 7.48	425.09 ± 16.37^a^	607.63 ± 9.10^a^	981.33 ± 84.27	529.44 ± 20.86^A^	669.56 ± 22.53^A^
	H CP	54.08 ± 2.96^A^	571.79 ± 13.94	131.27 ± 9.60	405.49 ± 32.63^b^	544.21 ± 23.45^b^	1021.33 ± 39.86	369.56 ± 53.96^B^	357.53 ± 23.15^B^
Additive Dosage	N-RES + HMB	26.67 ± 0.89	551.28 ± 18.89	154.39 ± 17.59	364.64 ± 16.87	666.43 ± 12.90^A^	1264.66 ± 23.09^b^	694.25 ± 89.82^a^	336.23 ± 10.32^B^
	RES+HMB	42.76 ± 10.17	606.08 ± 36.18	130.12 ± 12.62	481.63 ± 26.11	648.26 ± 39.45^B^	1494.66 ± 379.69^a^	451.20 ± 27.48^b^	355.67 ± 20.64^A^
*Protein*×*additives*	L	15.64 ± 12.11^a^	578.84 ± 12.43^ab^	142.25 ± 19.08	423.13 ± 67.02^b^	656.05 ± 30.46	1001.33 ± 62.90	449.50 ± 94.90	345.95 ± 18.07^B^
	H	29.35 ± 27.16^c^	578.68 ± 39.59^ab^	129.11 ± 8.15	415.29 ± 25.46^bc^	575.92 ± 38.20	1379.66 ± 271.56	555.37 ± 141.23	356.60 ± 19.64^B^
	L-RES + HMB	34.72 ± 10.92^b^	568.58 ± 22.79^a^	140.52 ± 19.41	385.06 ± 32.25^a^	637.03 ± 33.71	1123.00 ± 164.73	611.85 ± 107.47	502.90 ± 183.24^B^
	H-RES + HMB	48.23 ± 9.12^d^	588.94 ± 30.89^b^	130.85 ± 10.06	453.36 ± 36.59^c^	603.67 ± 63.67	1258.00 ± 354.28	416.21 ± 57.03	513.54 ± 172.12^A^
*P-*value	CP Level	0.000	0.145	0.214	0.001	0.022	0.267	0.000	0.000
	RES-HMB	0.121	0.990	0.104	0.587	0.000	0.010	0.002	0.000
	RES-HMB×CP level	0.001	0.026	0.076	0.009	0.159	0.425	0.998	0.000

Lactate dehydrogenase (LDH), Glucose (GLU), Muscle glycogen (MG), Hexokinase (HK), Pyruvate Kinase (PK), Creatine Kinase (CK), Malate Dehydrogenase (MDH), Succinate dehydrogenase (SDH), Lactic acid (LA). Data in the same column with the same or no lower case letters indicate nonsignificant differences (*p* > 0.05) and data with different lower case letters indicate significant differences (*p* < 0.05).

### 3.5 Effect on the liver transcriptome

**Figure 2:**
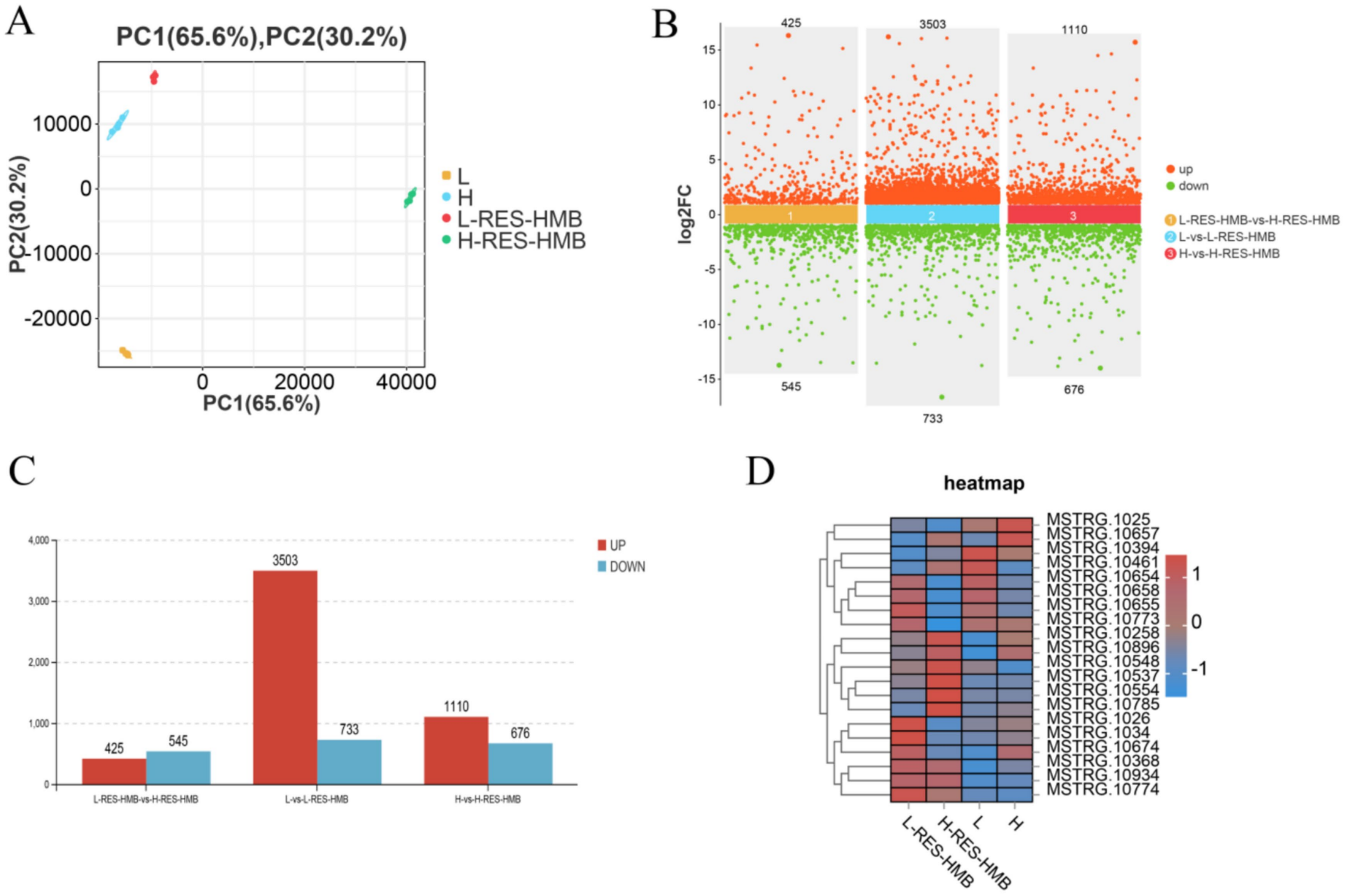
Transcriptome analysis of Tibetan sheep fed with different levels of resveratrol and HMB. **(A)** PCA analysis was conducted on samples from Group L, L-RES + HMB, H, and H-RES + HMB. Each dot in the graph represents an individual liver sample (*n* = 4 per group). A principal component analysis (PCA) reveals a clear separation between the groups, indicating distinct transcriptome characteristics between each group. **(B)** The differences in gene expression among the four groups are represented in a scatter plot. The *x*-axis represents the log_2_ fold change in gene expression between groups, while the *y*-axis shows the -log_10_ transformed adjusted *p*-value (Benjamini–Hochberg correction). **(C)** The number of upregulated and downregulated DEGs is depicted when comparing Group L to L-RES + HMB, Group H to H-RES + HMB, and Group L-RES + HMB to H-RES + HMB. **(D)** Heatmap analysis of the DEGs identified across the 16 sequenced libraries, with upregulated and downregulated genes displayed in red and blue, respectively.

Four liver tissue samples from each group (L, L-RES + HMB, H, and H-RES + HMB groups) were used to prepare RNA libraries. The RNA was sequenced using the Illumina HiSeq 2,500 platform. A total of 39.70 million, 39.01 million, 39.68 million, and 39.73 million raw reads were obtained for the L, L-RES + HMB, H, and H-RES + HMB libraries, respectively, with clean reads amounting to 39.47 million, 38.65 million, 39.32 million, and 39.34 million after filtering. Principal component analysis (PCA) of the clean data revealed good data repeatability across different samples (Figure 2A), indicating that the samples were suitable for downstream analysis. DEGs were identified based on standard criteria (log_2_(FC) > 1.5, *p <* 0.05) (Figure 2B). Among a total of 12,847 genes expressed in the livers of Tibetan sheep between the L and L-RES + HMB groups, ultimately, 4,236 DEGs were identified. These included 3,503 upregulated DEGs (82.70%) and 733 downregulated DEGs (17.30%) in the L-RES + HMB group (Figure 2C). The top 10 DEGs were ACOT12, LIPG, TPH1, TAX1BP1, PAICS, ACACA, FASN, LPIN1, PC, and UGP2. In groups H and H-RES + HMB, a total of 12,847 expressed genes were detected, with 1786 deGs were identified, including 1,110 degs (62.15%) and 676 degs (37.85%) in the H-RES + HMB group (Figure 2C). The top 10 DEGs were MT-ND4, PCK1, IL1R1, S1PR3, SDS, RPL21, TNC, FKBP5, GK, and HAL. In groups L-RES + HMB and H-RES + HMB, a total of 12,847 expressed genes were detected. Ultimately, 970 DEGs were identified, including 425 upregulated DEGs (43.18%) and 545 downregulated DEGs (56.19%) in the L-RES + HMB group (Figure 2C). The top 10 DEGs were S1PR3, GABRG1, JUN, TUBA1C, AIF1L, PHLDA1, AKR1C1, TNC, DNAJA1, and ENPP3. A heatmap-based comparison of DEG expression profiles across the four groups showed that the DEGs in the L, L-RES + HMB, H, and H-RES + HMB samples clustered separately, with good clustering of intragroup replicates (Figure 2D).

**Figure 3:**
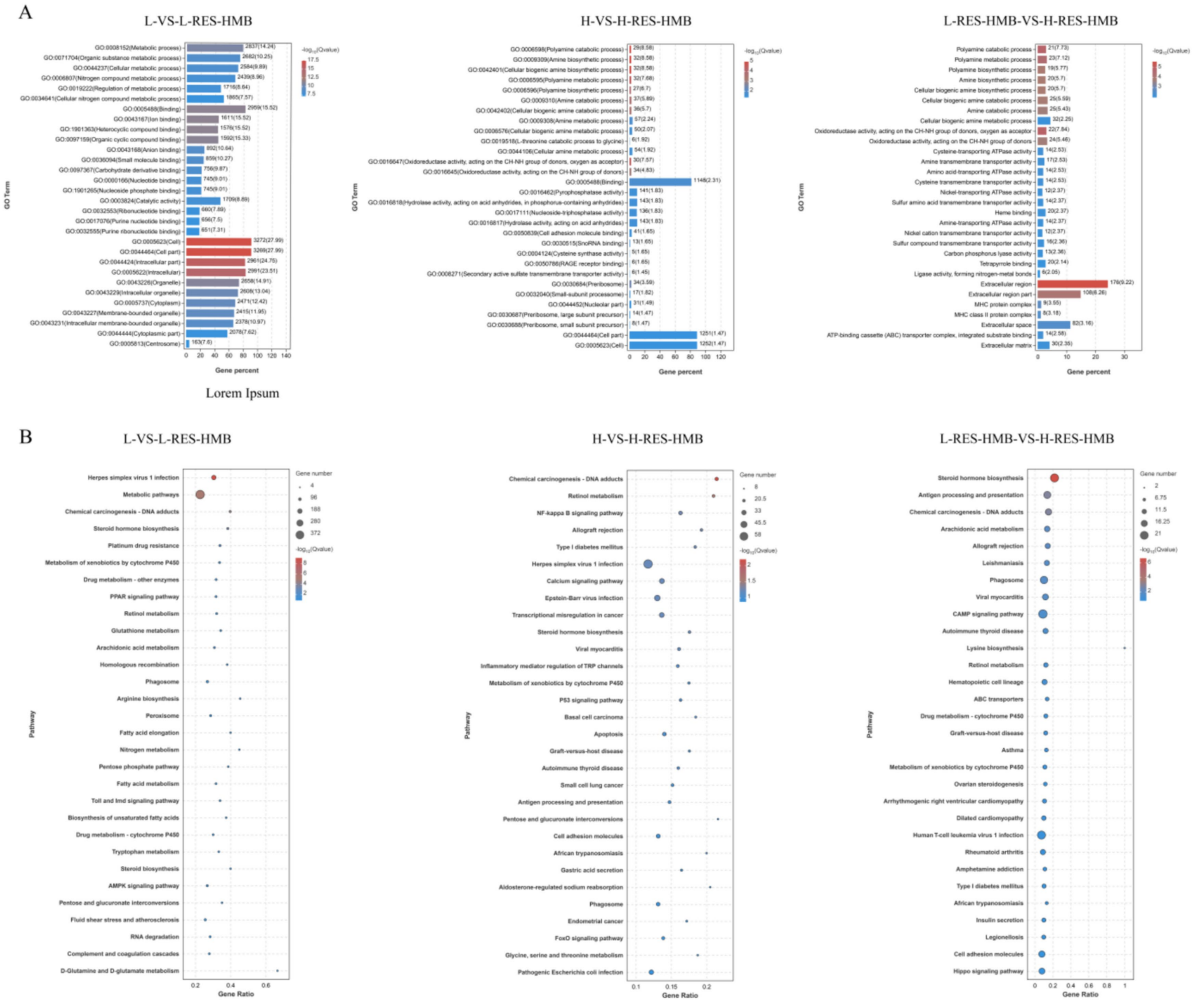
Functional enrichment analysis of Tibetan sheep fed with different levels of resveratrol and HMB. **(A)** GO enrichment bars of the enriched differential genes, with the ordinate indicating the GO terms. **(B)** Bubble plot of the top 20 KEGG pathways with ordinate indicating pathway.

Functional annotation of DEGs was performed via Gene Ontology (GO) and KEGG enrichment analyses. DEGs were predominantly associated with the following GO terms: extracellular region; amine biosynthetic process; oxidoreductase activity (acting on CH-NH group donors, oxygen as acceptor); polyamine catabolic process; and molecular binding (Figure 3A). These DEGs were also significantly enriched across 20 KEGG pathways (*p <* 0.05) (Figure 3B), including “Calcium signaling pathway (ko04020),” “NF-kappa B signaling pathway (ko04064),” “cAMP signaling pathway (ko04024),” and “PPAR signaling (ko03320),” which act as important regulators of immune function. Specifically, the NF-κB signaling pathway contained enriched DEGs including IGHG1, IGHV4-39, IL1R1, and TRB. Conversely, calcium signaling pathway enrichment featured LTB4R2, CACNA1S, CHRM3, PTGFR, TRB, HTR4, IGHV4-39, and IGHG1 (Figure 3B).

**Figure 4:**
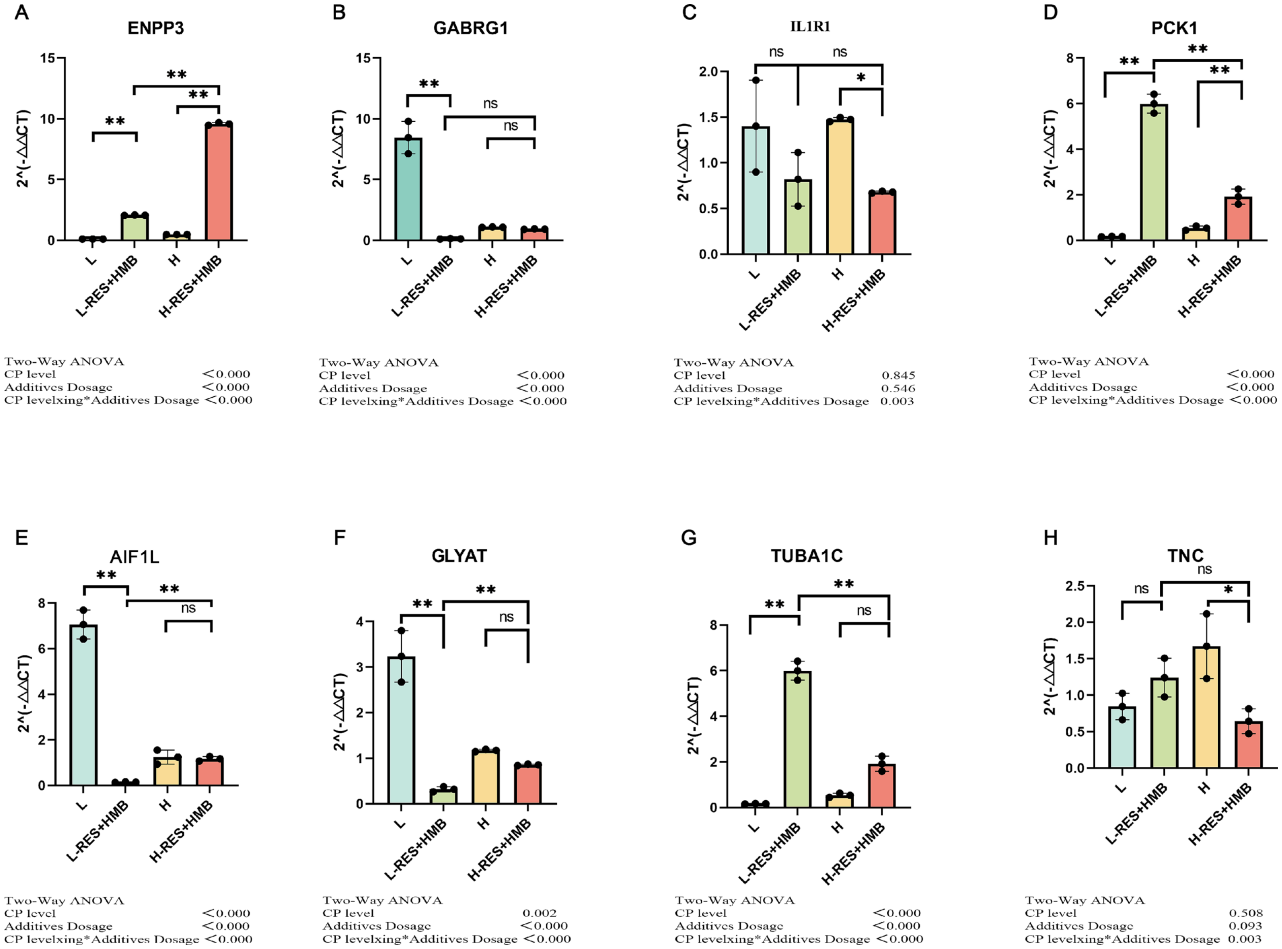
qRT-PCR analysis. qRT-PCR results were consistent with RNA-seq data. **(A)** ENPP3; **(B)** GABRG1; **(C)** IL1R1; **(D)** PCK1; **(E)** AIF1L; **(F)** GLYAT; **(G)** TUBA1C; **(H)** TNC. *This generally indicates a statistically significant difference at the *p* < 0.05 level. **This typically indicates a highly statistically significant difference at the *p* < 0.01 level. (not significant): This indicates that there was no statistically significant difference between the groups being compared.

To assess the reliability of the RNA-seq results, seven genes were selected at random, and their expression levels were verified using qRT-PCR. All mRNA levels were consistent with the RNA-seq data, indicating that the RNA-seq data were reliable (Figure 4).

### 3.6 Weighted gene co-expression network analysis

**Figure 5:**
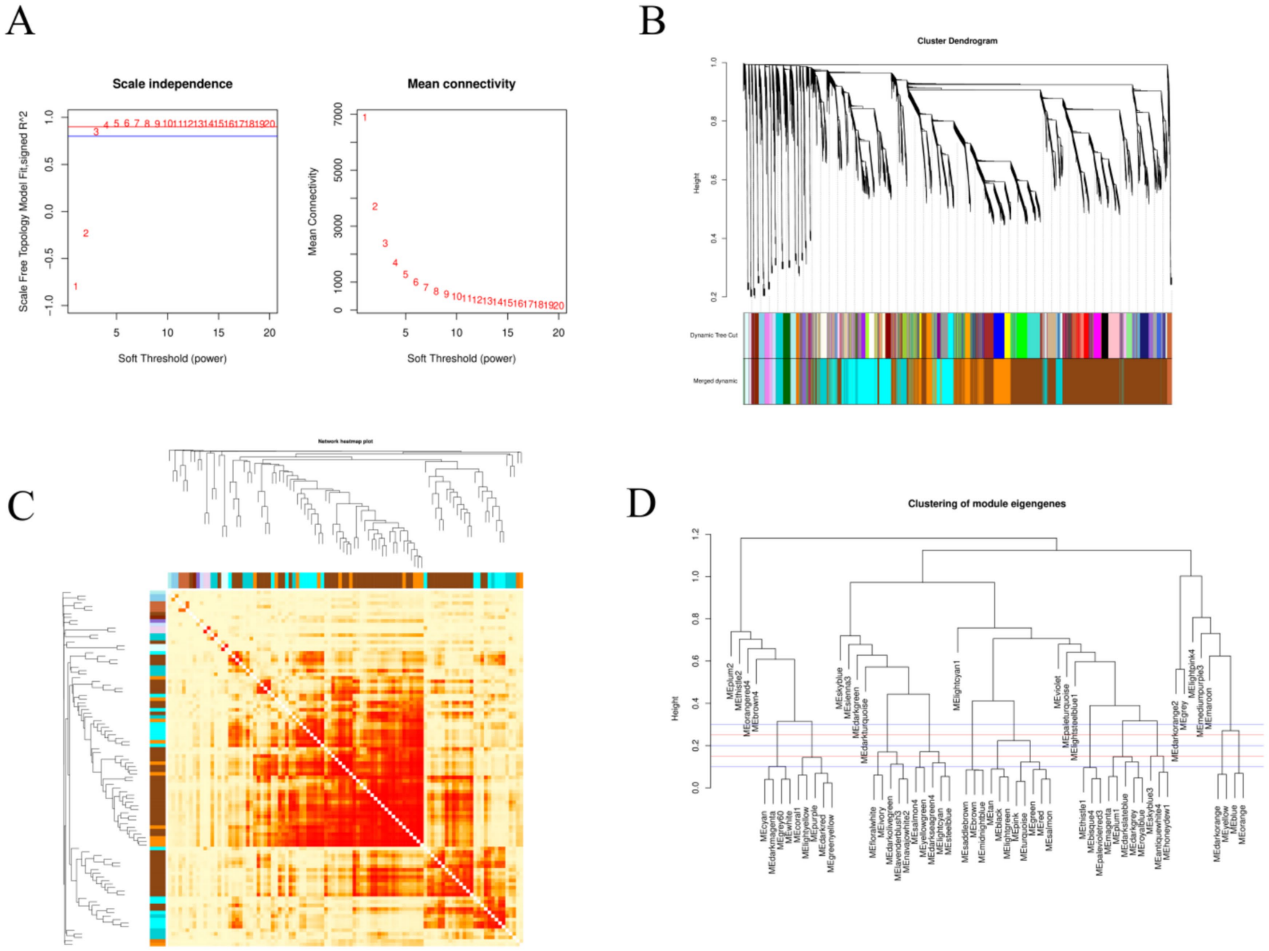
WGCNA analysis. **(A)** The left panel shows the scale-free fit index analysis for various soft thresholding powers, and the right panel shows the average connectivity analysis for various soft thresholding powers. **(B)** The RNA dynamic cutting cluster tree, where each color represents a module. The colors in the first row are the results of the initial clustering, and the colors in the second row are the results after module merging. **(C)** A heatmap of the co-expression module network (increasingly saturated red indicates a higher degree of overlap between functional modules). The rows and columns of the matrix represent different genes. **(D)** A module clustering diagram; the first red line is at a height of 0.25, and the modules below the red line are those that are more similar and need to be merged.

WGCNA was performed to analyze the 18,852 genes obtained after data preprocessing. A soft threshold of 4 was set based on a correlation threshold of 0.90 (Figure 5A). WGCNA was used to construct 36 co-expression modules (Figure 5B). After merging modules with similarity >0.50, 20 co-expression modules were obtained (Figures 5B,D). The majority of genes were enriched in the brown module (8,815 genes), followed by the blue module (2,988 genes), yellow module (2,462 genes), and green module (2003 genes). A Tree of Module Membership (TOM) cluster tree and TOM matrix heatmap were plotted based on gene expression levels to explore the interactions among the 20 co-expression modules (Figure 5C). Highly correlated genes exhibited tight clustering in high-intensity regions, confirming that co-modular genes share highly similar expression profiles.

**Figure 6:**
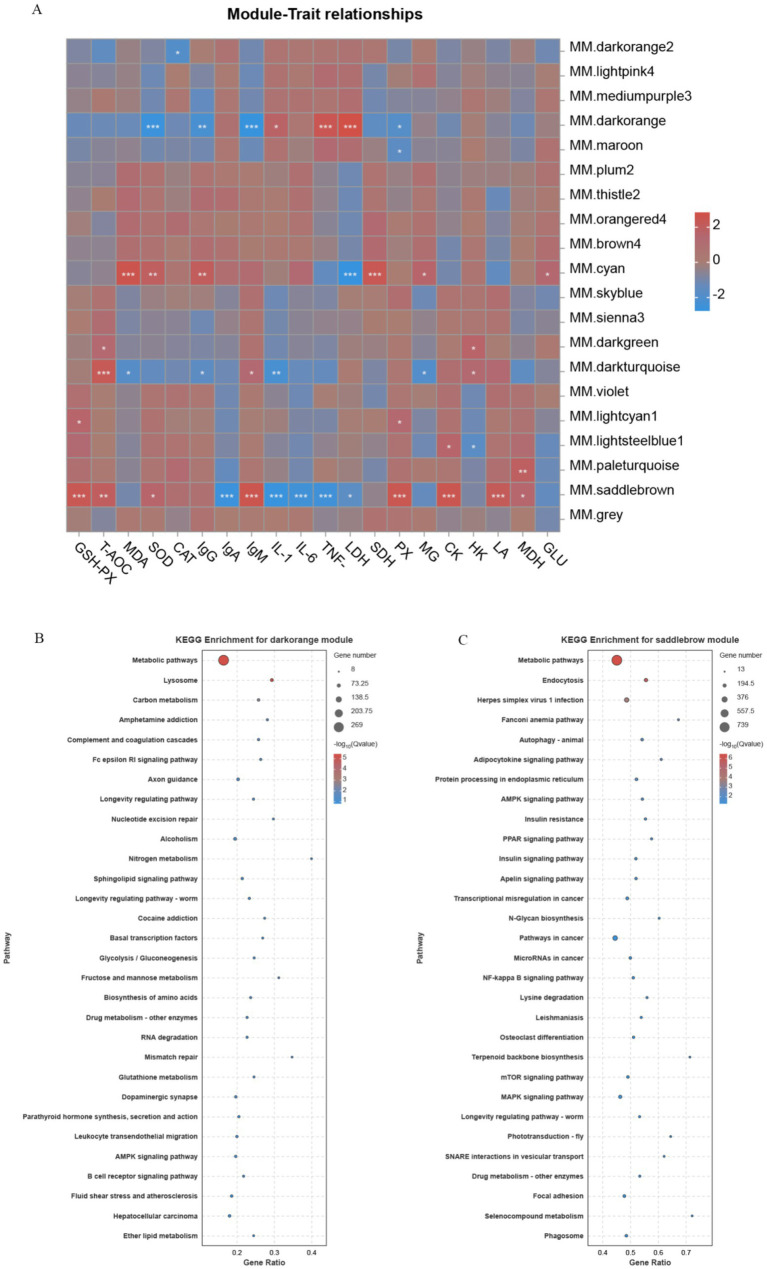
Correlation heatmap of module genes with traits and KEGG enrichment results. **(A)** The vertical coordinate on the right represents a set of co-expressed genes within each module; the darker the color in the heatmap, the closer the absolute value of the correlation coefficient is to 1, indicating that the gene module on the right is more closely related to the biological traits below. Panels **(B,C)** show the bubble charts of the top 20 KEGG pathway enrichments, with the vertical coordinate indicating the pathways.

Correlation heatmaps were constructed using weighted gene co-expression network analysis (WGCNA)-derived modules and quantified antioxidant, immune, and glycolytic indices (Figure 6). The results showed that the co-expressed genes in MM.saddlebrown had a highly significant correlation with the levels of GSH-PX, IgA, IgM, IL-1β, IL-6, TNF-*α*, PX, CK, and LA. Meanwhile, the co-expressed genes in MM.darkorange demonstrated a highly significant correlation with the levels of SOD, IgM, TNF-α, and LDH, whereas those in MM.cyan exhibited a highly significant correlation with the levels of MDA, LDH, and SDH. Through KEGG enrichment analysis of the genes in MM.saddlebrown and MM.darkorange, we found that MM.saddlebrown was enriched with signaling pathways related to antioxidant, immune, and glycolytic activities, including the AMPK signaling pathway, NF-kappa B signaling pathway, PPAR signaling pathway, and Selenocompound metabolism. Similarly, MM.darkorange was also enriched with signaling pathways related to antioxidant, immune, and glycolytic activities, including Glutathione metabolism, AMPK signaling pathway, Fc epsilon RI signaling pathway, B cell receptor signaling pathway, and Glycolysis/gluconeogenesis.

### 3.7 Metabolomics analysis of the liver

**Figure 7:**
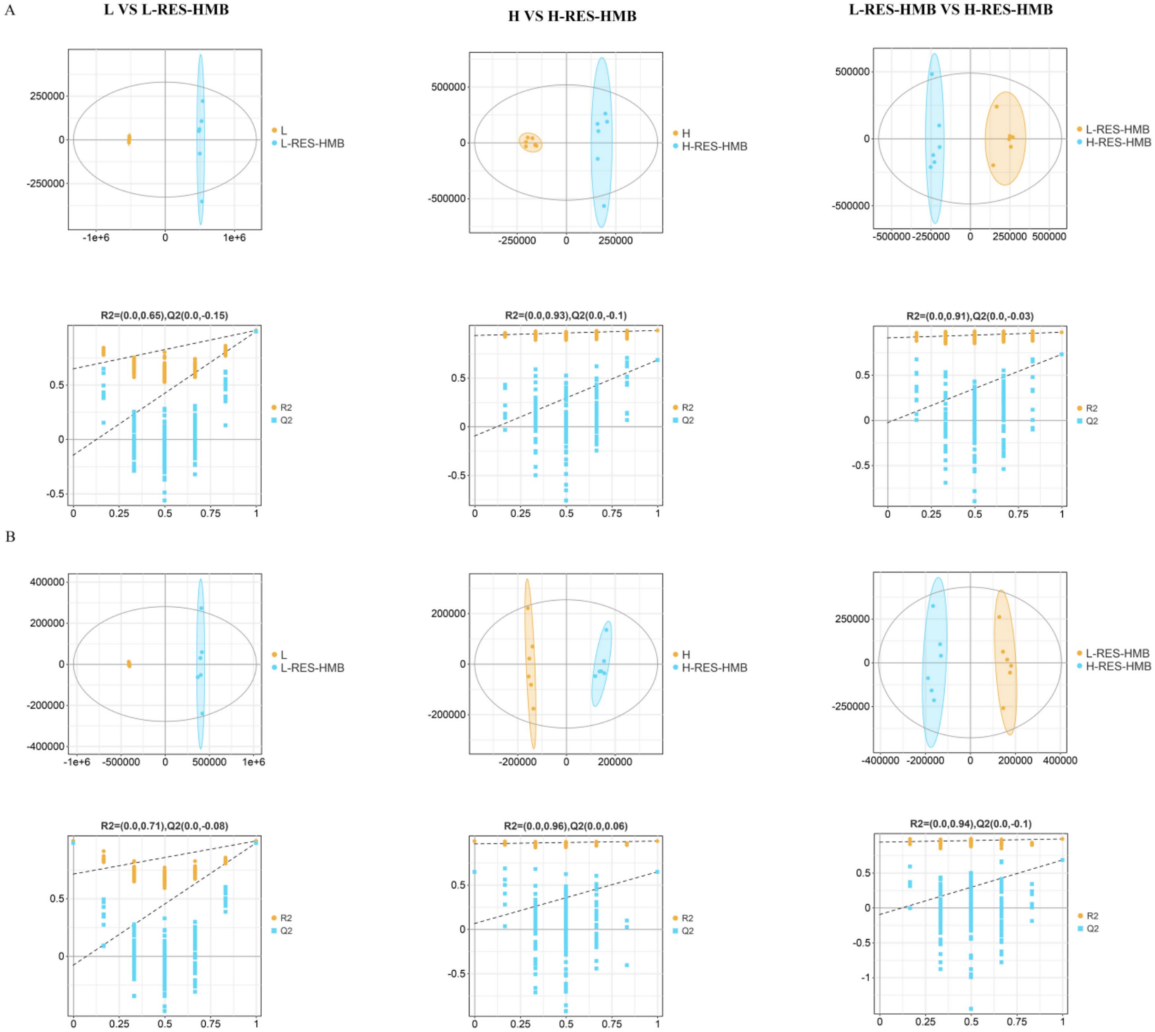
The level of liver metabolites of Tibetan sheep fed with different levels of resveratrol and HMB. **(A)** In positive ion mode, the OPLS-DA plots for Group L vs. L-RES-HMB, Group H vs. H-RES-HMB, and Group L-RES-HMB vs. H-RES-HMB are shown. The *x*-axis represents the predictive component score values, which are used to display the differences between groups, and the y-axis represents the orthogonal component score values, which are used to display the differences between groups. The second row shows the permutation test plots, which are used to assess the accuracy of the OPLS-DA, with the *y*-axis representing the *R*^2^*Y* or *Q*^2^ values and the *x*-axis representing the permutation retention rate. **(B)** In negative ion mode, the OPLS-DA plots for Group L vs. L-RES-HMB, Group H vs. H-RES-HMB, and Group L-RES-HMB vs. H-RES-HMB are shown. The rest is the same as in **(A)**.

LC–MS/MS was employed to explore the potential effects of different dietary protein levels combined with RES and HMB supplementation on hepatic metabolomics changes in Tibetan sheep. A total of 12,215 and 5,372 valid peaks were generated in the positive and negative ion modes, respectively. Partial least squares-discriminant analysis (OPLS-DA) score plots demonstrated good fitness (*R*^2^*X* and *R*^2^*Y*) and predictability (*Q*^2^) (Figure 7). In the positive ion mode, the *R*^2^*X*, *R*^2^*Y*, and *Q*^2^ values for L vs. L-RES-HMB, H vs. H-RES-HMB, and L-RES-HMB vs. H-RES-HMB were 0.811, 0.999, and 0.989; 0.4, 0.987, and 0.683; and 0.425, 0.973, and 0.729, respectively. Permutation tests for the OPLS-DA models revealed *R*^2^ and *Q*^2^ intercept values of 0.65 and −0.15, 0.93 and −0.1, and 0.91 and −0.03 for the L vs. L-RES-HMB, H vs. H-RES-HMB, and L-RES-HMB vs. H-RES-HMB, respectively, in the positive ion mode (Figure 7A). In the negative ion mode, the *R*^2^*X*, *R*^2^Y, and *Q*^2^ values for L vs. L-RES-HMB, H vs. H-RES-HMB, and L-RES-HMB vs. H-RES-HMB were 0.81, 0.999, and 0.981, 0.248, 0.993, and 0.646; and 0.396, 0.985, and 0.683, respectively. The *R*^2^ and *Q*^2^ intercepts in the negative ion mode were 0.71 and −0.08, 0.96 and 0.06, 0.94 and −0.01, respectively (Figure 7B).

**Figure 8:**
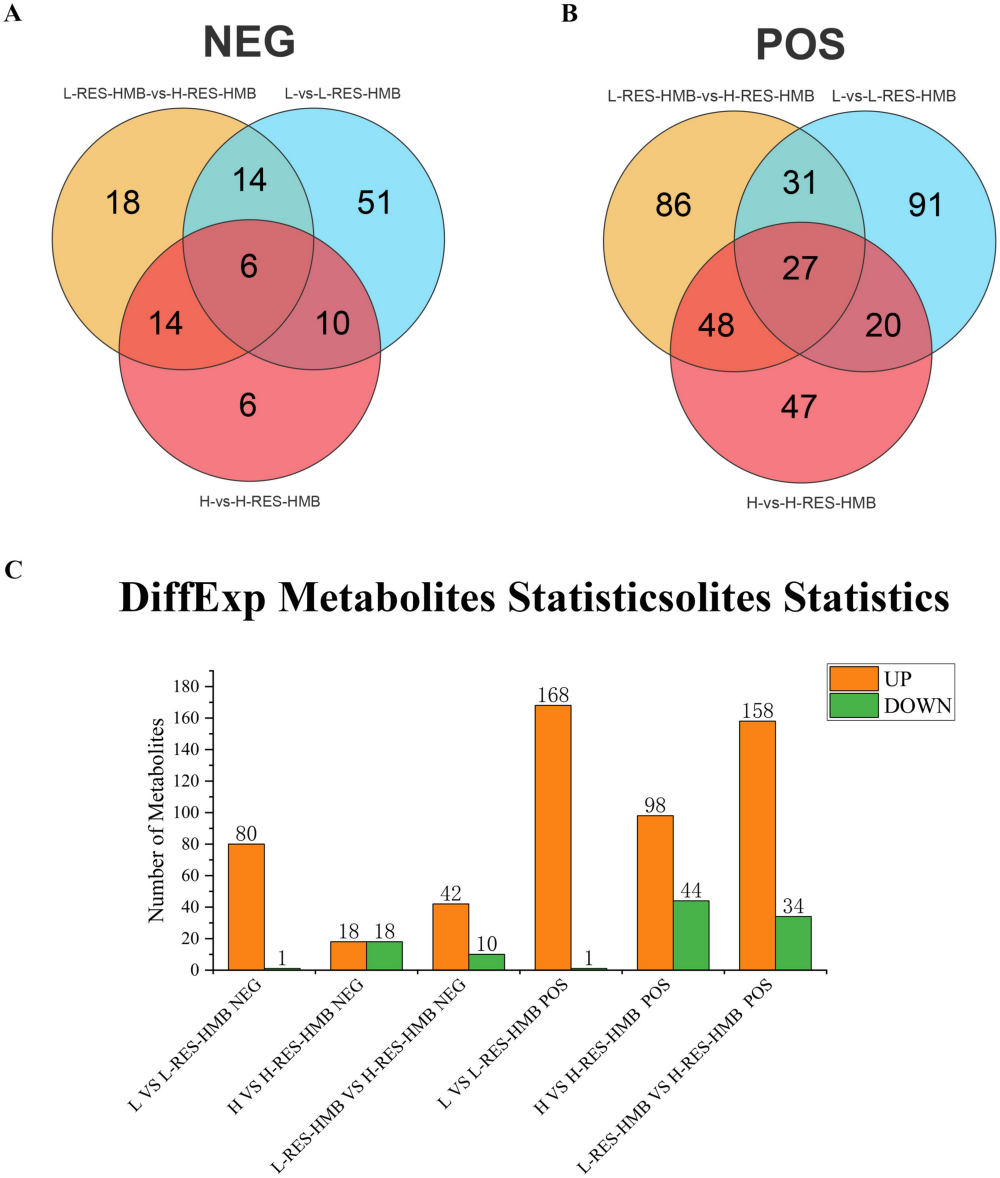
LC-MS/MS analysis of Tibetan sheep fed with different levels of resveratrol and HMB. **(A)** The Wayn diagram shows three comparisons (L versus L-RES-HMB; H versus H-RES-HMB; L-RES-HMB versus H-RES-HMB) NEG metabolites. **(B)** The En diagram shows three comparisons (L versus L-RES-HMB; H versus H-RES-HMB; L-RES-HMB versus H-RES-HMB) POS metabolites. **(C)** Number of up/down regulated metabolites in different comparison groups in POS and NEG modes.

Applying established DAM identification thresholds (VIP > 1, *p <* 0.05), we identified 892 and 366 KEGG-annotated metabolites in positive and negative ion modes, respectively (Figure 8). Pairwise comparisons revealed 250 DAMs between groups L and L-RES-HMB (169 positive, 81 negative mode), 178 DAMs between groups H and H-RES-HMB (142 positive, 36 negative mode), and 244 DAMs between groups L-RES-HMB and H-RES-HMB (192 positive, 52 negative mode). A comparison across all four groups in the positive ion mode revealed 27 common DAMs, which included 1,2-dihexadecanoyl-sn-glycero-3-phosphocholine, 3-amino-2,3-dihydrobenzoic acid, 4-amino-2,6-dinitrotoluene, argininosuccinic acid, benzoic acid, betaine, carbetamide, cycloheximide, dl-phenylalanine, dulcitol, guanine, harmaline, homatropine, isogentisin, isopropalin, Leu-Glu-Arg, Leu-His-Arg, *N*-acetyl-d-galactosamine, ononin, pro-pro, propranolol, and *S*-adenosyl-l-homocysteine. Negative ion mode analysis detected six common DAMs: α-d-galactose 1-phosphate, crotonoside, dl-serine, dl-threonine, dl-vanillylmandelic acid, and succinic semialdehyde (Figure 8).

**Figure 9:**
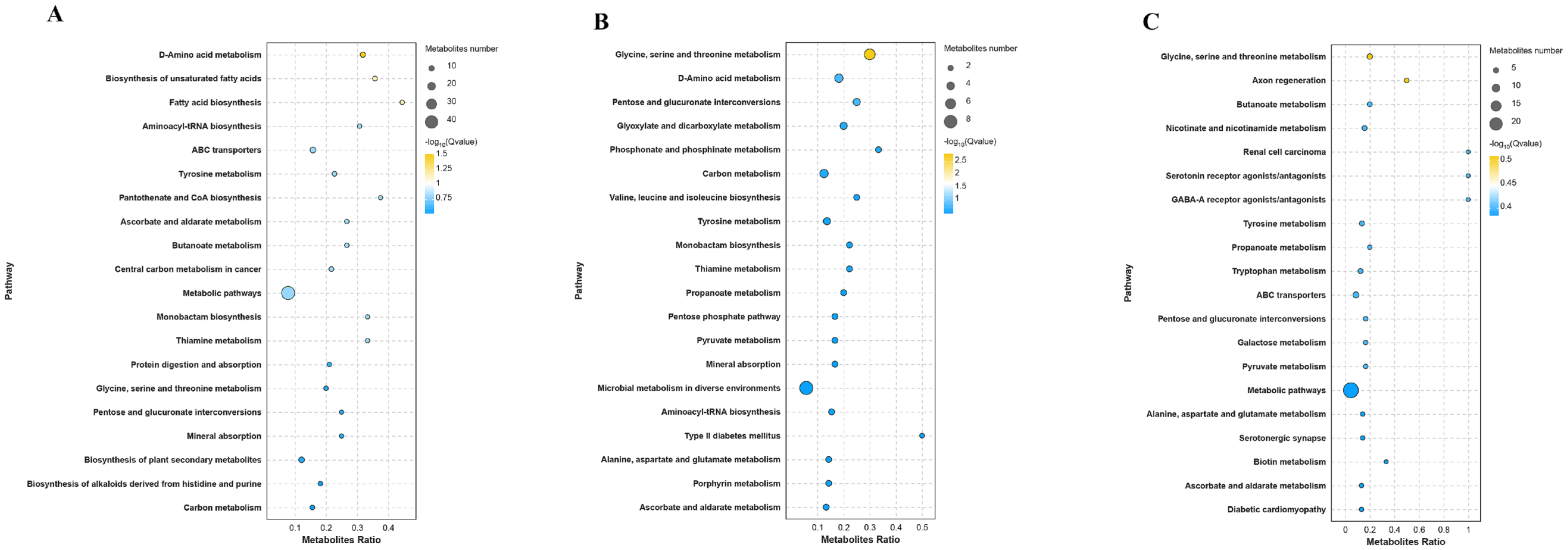
Metabolic pathway enrichment analysis of Tibetan sheep fed with different levels of resveratrol and HMB. In the metabolic pathway enrichment analysis, the size of the bubbles indicates the number of differential metabolites enriched in the pathways, and the color of the bubbles represents the significance of enrichment in the pathways, with larger values indicating more significant enrichment. **(A)** Enrichment analysis between Group L and L-RES-HMB. **(B)** Enrichment analysis between Group H and H-RES-HMB. **(C)** Enrichment analysis between Group L-RES-HMB and H-RES-HMB.

Metabolic pathway enrichment analysis elucidated functional associations of DAMs (Figure 9). DAMs in the L vs. L-RES-HMB comparison were predominantly enriched in glycine, serine and threonine metabolism and d-amino acid metabolism (Figure 9A). Similarly, H vs. H-RES-HMB DAMs exhibited primary enrichment in glycine, serine and threonine metabolism and d-amino acid metabolism (Figure 9B), while L-RES-HMB *vs*. H-RES-HMB DAMs showed significant enrichment in glycine, serine and threonine metabolism and ABC transporters (Figure 9C). Global comparison revealed DAM enrichment predominantly in biosynthesis of secondary metabolites.

### 3.8 Integrated metabolomics and transcriptomics analysis

**Figure 10:**
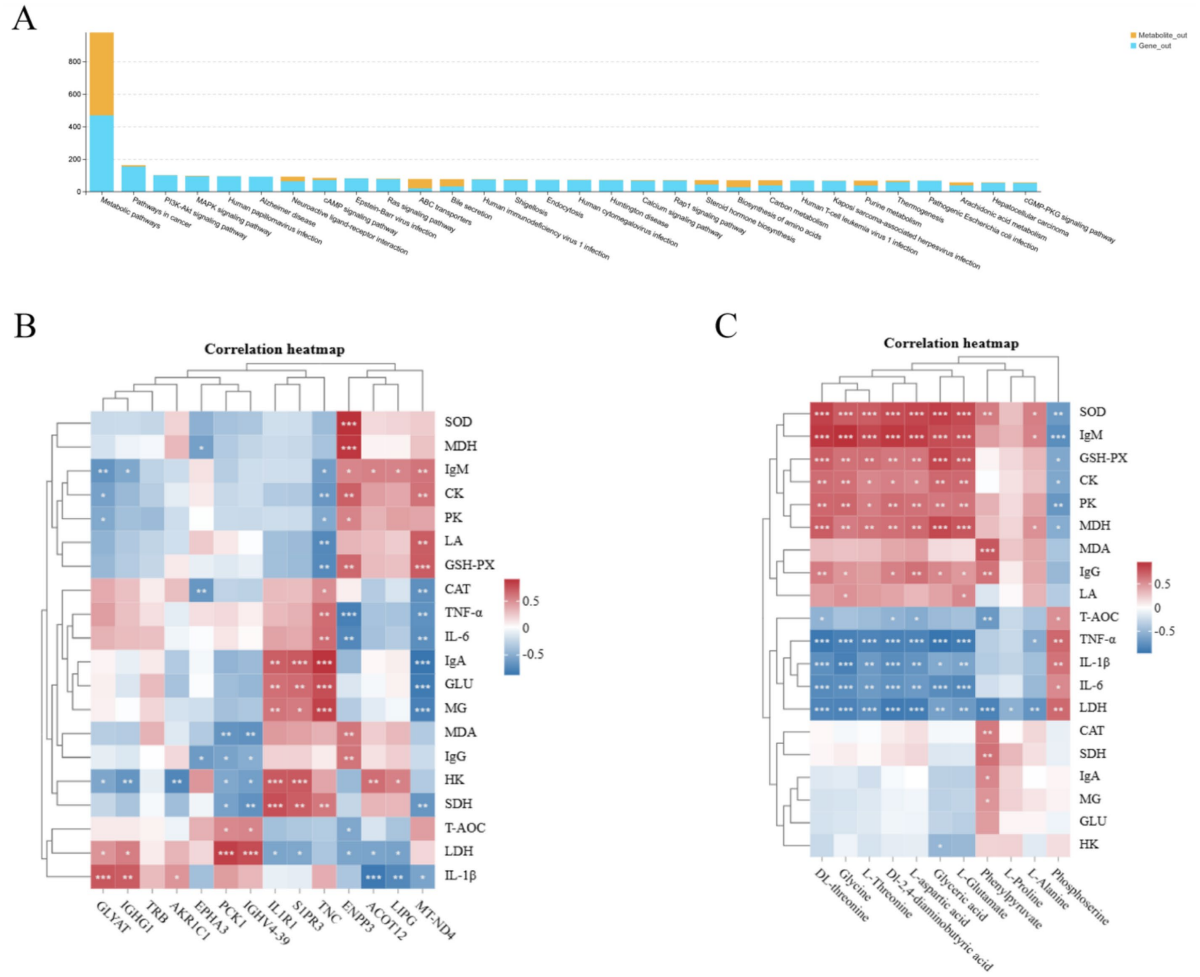
Correlation analysis of pathways, transcribed genes, and metabolites in Tibetan sheep fed with resveratrol and HMB. **(A)** Pathway correlation analysis histograms; **(B)** transcribed genes versus kit metrics heat map correlation analysis; **(C)** metabolites versus kit metrics heat map correlation analysis.

Integrated transcriptomic and metabolomic profiling provides a powerful approach for elucidating the molecular mechanisms underlying phenotypic variation. Transcriptome sequencing enables the identification of tissue-specific differential gene expression, while metabolomics delineates metabolic dynamics and associated regulatory pathways in biological systems. In this study, we integrated hepatic transcriptomic and metabolomic datasets to investigate the regulatory networks governing key phenotypic traits (Figure 10A). This integrative analysis revealed significant enrichment in several critical pathways, including Arachidonic acid metabolism, cAMP signaling, ABC transporters, and Carbon metabolism. Notably, within the arachidonic acid metabolism pathway-which modulates immune responses via the cyclooxygenase (COX) pathway-co-enrichment was observed for differentially expressed genes (DEGs; PGE2, ALOX5) and differentially abundant metabolites (DAMs; Lipoxin A4). Similarly, DEGs (ADCY10, PDE4B, ADCY1, ALOX5) and DAMs (Adenosine 5′-monophosphate) were co-enriched in the cAMP signaling pathway. This pathway regulates hepatic glycolipid metabolism by activating protein kinase A (PKA) and exchange protein directly activated by cAMP (EPAC), thereby modulating glycolytic activity. The relationship between DEGs and antioxidant, immune, and glycolytic indices showed that among the various antioxidant indexes, GSH-PX, SOD, and MDA were positively correlated with ENPP3 and MT-ND4, and negatively correlated with PCK1 and IGHV4-39. Among the immune indicators, IgM and IL-1β were positively correlated with GLYAT. Of the glycolytic indices, LDH was positively correlated with PCK1 and IGHV4-39, SDH was positively correlated with IL1R1, S1PP3, and TNC, and glucose and MG were positively correlated with TNC (Figure 10B). The relationship between DAMs and antioxidant, immune, and glycolytic indices was identified (Figure 10C), which the results showing that the metabolites dl-threonine, glyceric acid, glycine, l-threonine, dl-2,4-diaminobutyric acid, l-aspartic acid, and l-glutamate were positively correlated with SOD, IgM, GSH-PX, CK, and MDA activities.

## 4 Discussion

Transcriptomic and metabolomic analyses were conducted on hepatic tissues from Tibetan sheep to identify DEGs and DAMs following dietary supplementation with RES and HMB. Integrated omics analysis revealed distinct DEG and DAM profiles in Tibetan sheep administered RES and HMB. GO analysis indicated that RES and HMB supplementation predominantly modulated biological process and molecular function categories. KEGG pathway analysis was performed to elucidate biochemical and signal transduction pathways linked to DEGs and DAMs. Significantly enriched pathways included calcium signaling, cAMP signaling, NF-κB signaling, and PPAR signaling. qRT-PCR was employed to validate transcriptomics-identified DEGs and quantify their expression levels. Additionally, hepatic antioxidant, immune, and glycolytic indices were assessed to evaluate RES and HMB effects on transcriptomic and metabolomic profiles. Owing to Tibetan sheep’s unique high-altitude adaptations, the generalizability of these findings to low-altitude ruminants requires validation. Collectively, this study demonstrates beneficial effects of RES and HMB on hepatic health and metabolism in Tibetan sheep under differential dietary protein conditions. These findings provide novel strategies for optimizing animal nutrition and health. Further research should explore therapeutic applications across species and environments to advance sustainable animal husbandry.

The liver, a central metabolic organ, is encased by Glisson’s capsule—a connective tissue sheath (23, 24). It contains hepatocytes, liver sinusoids, hepatic veins, portal veins, and the biliary system (25, 26). Liver sinusoids are minute vascular channels within the hepatic lobules that receive blood from both the hepatic artery and the portal vein, thereby facilitating efficient exchange of substances with hepatocytes (27). Proteins are recognized as essential nutrients for maintaining liver health. Notably, the present study revealed that higher dietary protein levels promote hepatocyte regeneration and alleviate morphological damage in the liver tissue of Tibetan sheep. In particular, lambs fed a high-protein diet exhibited well-preserved liver histoarchitecture, characterized by plump and orderly hepatocytes. RES, a polyphenolic compound, can suppress nitric oxide production and bolster antioxidant defenses, thereby safeguarding liver tissue. Previous studies have also demonstrated that RES can attenuate acute alcohol-induced liver injury in rats (28). Furthermore, research has reported that the combined dietary supplementation of RES and HMB maintains normal hepatic morphology and enhances hepatocyte density (12). Consistent with these findings, the current study confirmed that the concurrent supplementation of RES and HMB exerts a beneficial effect on liver tissue development in Tibetan sheep.

Antioxidant capacity is crucial for the health and growth of livestock. The polyphenolic compound RES and HMB have been shown to exert significant antioxidant and immune-modulating effects. RES, a potent antioxidant predominantly found in grapes, mitigates oxidative stress in liver tissue by MDA levels and enhancing the activities of CAT and SOD (29). It also possesses anti-inflammatory properties, suppress the expression of pro-inflammatory cytokines such as TNF-*α* and IL-6, which are critical for hepatic inflammatory responses. Additionally, RES activates Kupffer cells in the liver, promoting hepatocyte regeneration and repair of damaged liver tissue (30). HMB, a metabolite derived from leucine, indirectly contributes to improved liver function by enhancing protein synthesis. Research indicates that HMB attenuates muscle protein degradation via the ubiquitin-proteasome pathway and promotes protein synthesis through activation of the mechanistic target of rapamycin (mTOR) signaling pathway, which may also alleviate oxidative stress (31–34). As a dietary supplement, HMB has demonstrated positive effects on immune response regulation in animals and is also known to modulate muscle protein catabolism via the phosphoinositide 3-kinase/protein kinase B (PI3K/Akt) signaling pathway (35). In the present study, lambs in the low-protein (L) group exhibited increased hepatic CAT activity and T-AOC, alongside decreased MDA levels. Conversely, lambs in the high-protein (H) group displayed enhanced SOD and GSH-PX activities, indicating that protein levels variably influence hepatic antioxidant defenses. Supplementation with RES and HMB (RES-HMB group) led to increased CAT, GSH-PX, and SOD activities and reduced MDA content in the liver. Notably, the high-protein RES-HMB group (H-RES-HMB) showed a particularly marked increase in SOD activity. These findings suggest that co-supplementation of RES and HMB under varying dietary protein conditions enhances hepatic antioxidant capacity by modulating the activities of key antioxidant enzymes, thereby supporting liver function and structural integrity. The liver also serves as a key immunological organ, defending against pathogens while maintaining immune homeostasis by balancing immune activation and tolerance (35, 36). In this study, groups receiving higher crude protein (CP) levels exhibited significantly increased IgG and IgM concentrations, coupled with reduced levels of TNF-*α* and IL-6. Consistent with this, Yengkokpam et al. (37) reported that high-protein diets improve disease resistance and immune status in Nile tilapia fry. The H-RES-HMB group exhibited significantly elevated IgM levels and a notable reduction in TNF-α and interleukin-1β (IL-1β), in line with previous findings on the immunomodulatory effects of RES and HMB (12, 38). As discussed above, RES-HMB synergistically increases antioxidant capacity (elevated CAT/SOD and reduced MDA) and immune function (elevated IgM and reduced TNF-α/IL-6), especially when combined with a high-protein diet. This could mitigate oxidative stress in harsh plateau conditions and improve survival rates.

In this study, we examined the effects of RES and HMB on the activity of liver metabolic enzymes under different protein conditions. The H-RES-HMB group exhibited increased activities of glucose and hexokinase, indicating enhanced glycolytic capacity. In contrast, the high-protein control group (HPC) showed increased lactate dehydrogenase (LDH) and succinate dehydrogenase (SDH) activity, indicating a shift toward anaerobic glycolysis and the tricarboxylic acid (TCA) cycle, respectively. The low-protein control group (LPC) showed increased hexokinase, pyruvate dehydrogenase, malate dehydrogenase, and SDH activities, indicating balanced engagement in glycolysis and the TCA cycle. Glycolysis is a fundamental metabolic process that converts glucose into pyruvate, releasing energy to support cellular functions (39). Previous studies have suggested that RES enhances hepatic glycolytic capacity by activating the AMP-activated protein kinase (AMPK) pathway, thereby contributing to glucose homeostasis (40–43). Additionally, RES has been shown to modulate the mTOR signaling pathway, inhibiting glycolysis and proliferation in colorectal cancer cells (44). Dietary RES supplementation has also been shown to reduce LDH and lactate (LA) levels in animal models (45). Although the effect of HMB on liver metabolism remains unclear, studies indicate that HMB is internalized by cells via monocarboxylate transporters that co-transport H^+^ and are also involved in lactate and β-hydroxybutyrate transport (46–48). The H^+^-coupled uptake of HMB can lead to alterations in glycolysis, indicating that HMB may indirectly influence hepatic sugar metabolism by modulating related transport processes. The increased hexokinase activity observed in the H-RES-HMB group may be due to the synergistic effect of RES and HMB on glycolytic initiation. This effect could potentially be caused by enhanced glucose uptake and phosphorylation. High-protein diets with RES-HMB increased glycolytic enzymes (e.g., hexokinase) and metabolic flexibility. Farmers could use this information to improve feed efficiency and reduce protein waste while maintaining growth.

Transcriptomic analysis is a powerful approach for investigating alterations in gene expression. In this study, transcriptomic profiling elucidated the complex hepatic gene expression patterns in Tibetan sheep subjected to various dietary conditions, particularly after supplementation with resveratrol (RES) and β-hydroxy β-methylbutyrate (HMB) under different protein levels. Dietary protein intake directly regulates gene expression by affecting the efficiency of transcription and translation processes. Previous studies have demonstrated that low-protein conditions can constrain the expression of specific genes due to insufficient transcriptional activators, whereas high-protein intake facilitates gene expression (49–51). This finding indicates that the availability of proteins plays a pivotal role in the modulation of hepatic gene expression. RES and HMB affect gene expression via distinct regulatory mechanisms. Evidence suggests that RES may modulate gene transcription by activating the AMPK pathway, thereby impacting genes associated with hepatic metabolism (52, 53). For example, RES and HMB supplementation under low-protein conditions can alter the expression of hepatic genes, such as IL1R1 (decrease from 78.33 to 25.96). This potentially occurs due to the interaction between low protein levels and these additives, which influence the signaling pathways involving IL1R1 and consequently affect gene expression. Under high-protein conditions, the addition of RES and HMB induces distinct gene expression patterns, such as elevated expression levels of ATP2B2 (from 1.26 to 2.87), IGHG1 (from 53.58 to 188.33), and IGHV4-39 (from 11.73 to 68.60). The calcium signaling pathway and NF-κB signaling pathway mediate these effects. The calcium signaling pathway, a pivotal intracellular signaling cascade, is influenced by protein levels, RES, and HMB (54, 55). Variations in protein levels alter intracellular calcium ion concentrations, and the concurrent addition of RES and HMB may further modulate the activity of calcium ions and associated transport proteins. ATP2B2, which encodes a plasma membrane calcium ATPase (56), modulates the transmembrane transport of calcium ions, thereby influencing gene expression within the calcium signaling pathway (57). The NF-κB signaling pathway plays a crucial role in liver immunity (58, 59). Across varying dietary protein levels, the addition of RES and HMB modulates the NF-κB signaling pathway, influencing immune cell activation and inflammatory factor expression. IL1R1, a key protein for immune responses, is central to the NF-κB signaling pathway. Studies have shown that the binding of IL-1 to IL1R1 activates downstream signaling molecules, releasing NF-κB and promoting the expression of inflammatory genes (60). Elevated Ca^2+^ levels can activate calmodulin (CaM) and calmodulin-dependent protein kinase (CaMK), potentially influencing NF-κB activity (61). In summary, several DEGs involved in the calcium signaling and NF-κB pathways showed significant expression changes in the H-RES-HMB group and were closely linked to phenotypic observations. While the regulatory effects of RES on the AMPK and mTOR pathways are well established, the specific role of HMB in modulating calcium transport via monocarboxylate transporters and its interaction with the NF-κB pathway warrants further investigation.

Metabolomic analysis elucidated the impact of dietary protein levels and the combined supplementation of RES and HMB on hepatic metabolites and metabolic pathways in Tibetan sheep. A total of 250 differentially abundant metabolites (DAMs) were identified between the low-protein (L) and low-protein with RES and HMB (L-RES-HMB) groups, 178 between the high-protein (H) and high-protein with RES and HMB (H-RES-HMB) groups, and 244 between the L-RES-HMB and H-RES-HMB groups. Notably, dl-threonine, Glyceric acid, Glycine, and l-glutamate emerged as key DAMs in the latter two groups. dl-threonine, an essential amino acid, is critical for protein synthesis and immune modulation (62). Glyceric acid, a key intermediate in glycerol metabolism, plays a central role in energy production. Glycine, a non-essential amino acid, is involved in collagen biosynthesis and functions as a neurotransmitter influencing diverse physiological processes. l-glutamate, the primary excitatory neurotransmitter in the central nervous system, also serves as a precursor for glutamine synthesis, contributing to neurotransmitter balance and metabolic homeostasis (63). KEGG pathway enrichment analysis showed that these DAMs were predominantly involved in glycine, serine, and threonine metabolism, butanoate metabolism, pyruvate metabolism, and ATP-binding cassette (ABC) transporters. These results indicate that dietary protein levels, in combination with RES and HMB supplementation, significantly influence hepatic amino acid metabolism in Tibetan sheep. The liver, as the principal site for amino acid catabolism and utilization in ruminants, plays a vital role in systemic metabolic regulation (64). Changes in dietary protein levels modulate the activity of key metabolic enzymes, thereby altering DAM abundance and the activity of associated pathways. For example, the conversion of threonine to glycine and serine is regulated by enzymes in the glycine, serine, and threonine metabolism pathway, which can be affected by protein intake (65). RES and HMB exert regulatory influences on hepatic metabolite levels and associated pathways through distinct mechanisms. RES can reprogram cellular metabolic states by regulating the expression of genes involved in metabolite biosynthesis. For instance, RES modulates the expression of serine hydroxymethyltransferase (SHMT), thereby altering glycine and serine metabolic flux. Although the direct effects of HMB on hepatic metabolism are not fully elucidated, HMB is believed to impact metabolism by altering cellular transport and intracellular signaling. It may interact with membrane transporter proteins or metabolic enzymes, thereby influencing metabolite uptake, transformation, and distribution. In the ABC transporter pathway, for example, HMB may indirectly modulate transporter function and subsequently affect hepatic metabolite profiles. Butanoate metabolism, pyruvate metabolism, and ABC transporter pathways are crucial for maintaining cellular energy homeostasis and metabolic equilibrium in animals (66). This study demonstrated that RES and HMB supplementation, when administered under varying dietary protein levels, exerted synergistic effects on the modulation of DAMs and KEGG metabolic pathways. Under low-protein conditions, RES and HMB may activate specific signaling pathways to counteract the effects of protein deficiency on liver metabolism, maintaining the levels of key metabolites and stabilizing threonine levels by modulating the activity of threonine dehydrogenase (TDH). Conversely, under high-protein conditions, RES and HMB may help prevent metabolic dysregulation by fine-tuning pathway activity (63). These modulatory effects were reflected in altered metabolite abundances, particularly within glycine, serine, and threonine metabolism, butanoate metabolism, pyruvate metabolism, and ABC transporter pathways. For instance, dl-threonine and l-threonine levels were notably reduced in the L-RES-HMB group compared to the H-RES-HMB group, highlighting the interaction between protein level and bioactive compound supplementation in shaping hepatic metabolism.

Our integrated transcriptomic and metabolomic analyses revealed that differentially expressed genes (DEGs), including PGE2 and ALOX5, alongside differentially abundant metabolites (DAMs) such as dl-threonine and glyceric acid, were predominantly enriched in immune signaling, glycolysis, arachidonic acid metabolism, and cAMP signaling pathways. These phenotypes and pathways exhibit significant interconnectivity. Immune function, glycolysis, and arachidonic acid metabolism exert co-regulatory effects on the cAMP signaling pathway. The liver functions as a pivotal immune organ, modulating immune responses through Kupffer cells, liver sinusoidal endothelial cells, and other immune components. The cAMP signaling pathway is essential for immune cell activation, proliferation, and differentiation, thereby influencing hepatic immune tolerance and inflammatory processes. Previous studies have demonstrated that cAMP signaling facilitates T and B cell activation and proliferation, regulates natural killer cell and macrophage functions, and plays a critical role in maintaining hepatic immune homeostasis (67, 68). Collectively, these immune regulatory networks sustain immune balance within the liver microenvironment. Moreover, the cAMP signaling pathway is integral to hepatocyte glycolysis regulation. Glycolytic control is crucial for liver immunity because it governs energy metabolism and functional capacities of immune cells. It is well established that immune cells experience increased energy demands during inflammation, and inhibition of glycolysis can significantly alter immune cell functions, thus impacting the overall immune equilibrium in the liver (69, 70). cAMP activates protein kinase A (PKA), which suppresses glycolysis while promoting gluconeogenesis, thus modulating hepatocyte responses during inflammatory stress (71). Furthermore, cAMP stimulates the release of arachidonic acid, and glycolytic metabolites can reciprocally influence the cAMP signaling pathway. Inflammatory lipid mediators derived from arachidonic acid metabolism, including prostaglandins and leukotrienes, provide critical feedback regulation of the cAMP pathway. These mediators interact with membrane receptors on immune cells, activating or inhibiting downstream signaling cascades, thereby modulating immune cell activity and function (72). This modulation of the cAMP pathway fine-tunes hepatic immune responses by altering cytokine secretion profiles in immune cells, thus preserving the stability of the hepatic microenvironment (73). In summary, the cAMP signaling pathway is critical for hepatic immune regulation by modulating immune function, glycolysis, and arachidonic acid metabolism. These processes interact reciprocally to improve liver health and immune function. While this study examined the cAMP pathway, further research is warranted to elucidate the effects of RES and HMB supplementation at varying protein levels on the liver.

## 5 Conclusion

In this study, the addition of RES and HMB to HP diets could alleviate oxidative stress in the liver by regulating the expression of genes and metabolites through the cAMP signaling pathway and enhancing hepatic immunoreactivity. Therefore, the addition of RES and HMB at a dietary crude protein content of 14% could increase the immunoreactivity of Tibetan sheep liver and reduce decrease oxidative stress without negatively affecting the liver development. However, these findings, despite demonstrating the synergistic effects of dietary protein levels and RES and HMB, further studies are needed to determine their long-term effects on growth performance, and meat quality, and to provide a theoretical basis for the development of Tibetan sheep farming.

## Data Availability

Publicly available datasets were analyzed in this study. The datasets presented in this study can be found in online repositories. The names of the repository/repositories and accession number(s) can be found: NCBI SRA (accession: PRJNA1122402), MetaboLights (accession: MTBLS10767).

## Footnotes

^1^ https://daehwankimlab.github.io/hisat2/
